# The advantages of SNP arrays over CGH arrays

**DOI:** 10.1186/1755-8166-7-S1-I31

**Published:** 2014-01-21

**Authors:** Boris Keren

**Affiliations:** 1 Genetic Department, La Pitié-Salpêtrière Hospital, AP-HP, Paris, France

## 

In recent years, with the rapid development of Chromosomal Micro-array Analysis (CMA), the resolution limit of 5Mb which was imposed by conventional cytogenetics, has been significantly lowered. Currently, array CGH is the most widely used CMA technology. With the inclusion of thousands to millions of probes, it allows the detection of small copy number variations (CNVs) of a few kb. SNP (Single Nuleotide Polymorphism) arrays can also be used for CMA. They too enable the detection of CNVs, but unlike array CGH, each probe is located at an SNP and can determine the genotype of the corresponding SNP.

Here, we report on our 6 years experience of the use of SNP arrays in cytogenetic diagnosis on more than 3000 patients and we will focus on the main benefits of SNP arrays over array CGH. Because of their ability to perform SNP genotyping, SNP arrays can detect long contiguous stretches of heterozygosity (LCSH).

LCSH have 2 main interests:

1) they can detect uniparental isodisomies (UPD);

2) they can detect genetic identity by descent.

UPD can be responsible for imprinting disorders and both UPD and identity by descent is associated withpromote the occurrence of autosomal recessive disorders. Moreover LCSH analysis allows performing homozygosity mapping and helping guide sequencing of candidate genes responsible for recessive conditions. Because of an abnormal number of different alleles, SNP arrays also enable the detection of polyploïdy and chimerism. Besides, SNP arrays also are of interest in quality control: they can detect DNA contamination and false paternity.

Thus, while array CGH is still a very efficient technique to detect CNVs, the inclusion of SNP probes in arrays is desirable when possible.

